# K_2P_2.1 (TREK-1) potassium channel activation protects against hyperoxia-induced lung injury

**DOI:** 10.1038/s41598-020-78886-y

**Published:** 2020-12-15

**Authors:** Tatiana Zyrianova, Benjamin Lopez, Riccardo Olcese, John Belperio, Christopher M. Waters, Leanne Wong, Victoria Nguyen, Sriharsha Talapaneni, Andreas Schwingshackl

**Affiliations:** 1 Department of Pediatrics, University of California Los Angeles, 10833 Le Conte Ave, MDCC 12-475, Los Angeles, CA 90095 USA; 2 Department of Anesthesiology and Perioperative Medicine, University of California Los Angeles, Los Angeles, CA USA; 3 Department of Physiology, University of California Los Angeles, Los Angeles, CA USA; 4 Department of Pulmonary and Critical Care Medicine, University of California Los Angeles, Los Angeles, CA USA; 5 Department of Physiology, University of Kentucky, Lexington, KY USA

**Keywords:** Cell biology, Mechanisms of disease

## Abstract

No targeted therapies exist to counteract Hyperoxia (HO)-induced Acute Lung Injury (HALI). We previously found that HO downregulates alveolar K_2P_2.1 (TREK-1) K^+^ channels, which results in worsening lung injury. This decrease in TREK-1 levels leaves a subset of channels amendable to pharmacological intervention. Therefore, we hypothesized that TREK-1 activation protects against HALI. We treated HO-exposed mice and primary alveolar epithelial cells (AECs) with the novel TREK-1 activators ML335 and BL1249, and quantified physiological, histological, and biochemical lung injury markers. We determined the effects of these drugs on epithelial TREK-1 currents, plasma membrane potential (Em), and intracellular Ca^2+^ (iCa) concentrations using fluorometric assays, and blocked voltage-gated Ca^2+^ channels (Ca_V_) as a downstream mechanism of cytokine secretion. Once-daily, intra-tracheal injections of HO-exposed mice with ML335 or BL1249 improved lung compliance, histological lung injury scores, broncho-alveolar lavage protein levels and cell counts, and IL-6 and IP-10 concentrations. TREK-1 activation also decreased IL-6, IP-10, and CCL-2 secretion from primary AECs. Mechanistically, ML335 and BL1249 induced TREK-1 currents in AECs, counteracted HO-induced cell depolarization, and lowered iCa^2+^ concentrations. In addition, CCL-2 secretion was decreased after L-type Ca_V_ inhibition. Therefore, Em stabilization with TREK-1 activators may represent a novel approach to counteract HALI.

## Introduction

Oxygen supplementation (hyperoxia; HO) is the most frequently administered therapy in hospitalized patients and the mainstay of treatment for hypoxic respiratory failure, regardless of its etiology^1^. Clinically, supra-physiologic levels of oxygen tension are often tolerated and perceived as a safety net against hypoxemia^2^. As a result, in the US each year approximately 800,000 patients are exposed to HO therapy at a cost of $1.8 billion to the health care budget^3^. Importantly, the degree and duration of HO exposure positively correlate with patient morbidity and mortality rates^4–6^.

Although oxygen therapy can be a life-saving intervention, ample experimental and clinical evidence demonstrates that excessive levels of oxygen supplementation can also initiate and accelerate existing lung injury (HO-induced acute lung injury; HALI). Animal models of HALI have been particularly helpful in investigating the underlying mechanisms^7^, and studies in healthy adults showed that HO exposure causes tracheobronchitis and changes in vital capacity, diffusing capacity, and lung permeability within only six hours, and with a severity that is proportional to the degree of HO exposure^8–13^. Experimentally, a similar dose- and time-dependent inflammatory response to HO can be reproduced in animal models of HALI^14–16^, demonstrating close similarities in lung injury phenotypes between animals and humans^15,17–20^. From these studies we learned that the molecular mechanisms underlying HALI are complex and include extensive alterations in inflammatory cytokine secretion^14,21,22^. Both alveolar epithelial and endothelial cells are injured by HO, but the epithelial layer is the first line of defense against inhaled HO^23–25^.

Currently, minimizing the duration and amount of HO exposure of patients (“permissive hypoxemia”) represents the only clinical approach to limit HALI, and so far no molecular targets have been identified that translate into improved patient outcomes^26^. However, minimizing HO exposure is complicated by the lack of consensus in defining the lower limits of permissive hypoxemia, which would allow us to clinically differentiate beneficial from injurious levels of HO therapy^27,28^.

In our search for new molecular targets against HALI, we recently identified epithelial K_2P_2.1 (TREK-1) K^+^ channels as important regulators in the development and progression of HALI^29–32^. TREK-1 channels belong to the family of 2-pore domain (K2P) K^+^ channels, which are generally known for their unusual gating properties leading to so-called “leak K^+^ currents” that stabilize the resting plasma membrane potential (Em)^33,34^. In general, K2P channels, including TREK-1, are widely expressed in body tissues^35–41^, but their role in the lung remains largely unknown. Using in vivo and in vitro models of HALI, we previously discovered that HO exposure decreases the expression of TREK-1 channels in mouse lungs and alveolar epithelial cells, and accelerates alveolar inflammation and barrier dysfunction^30,42,43^. These findings sparked the hypothesis that despite HO-mediated TREK-1 downregulation, pharmacological activation of the remaining subset of TREK-1 channels can protect against HALI. To test this hypothesis, in this study we explored the potential protective effects and underlying mechanisms of two novel TREK-1 activating compounds (ML335, BL1249) using in vivo and in vitro models of HALI.

## Results

### Intra-tracheal administration of TREK-1 activating compounds protects mice against HO-induced acute lung injury (HALI)

**Figure 1:**
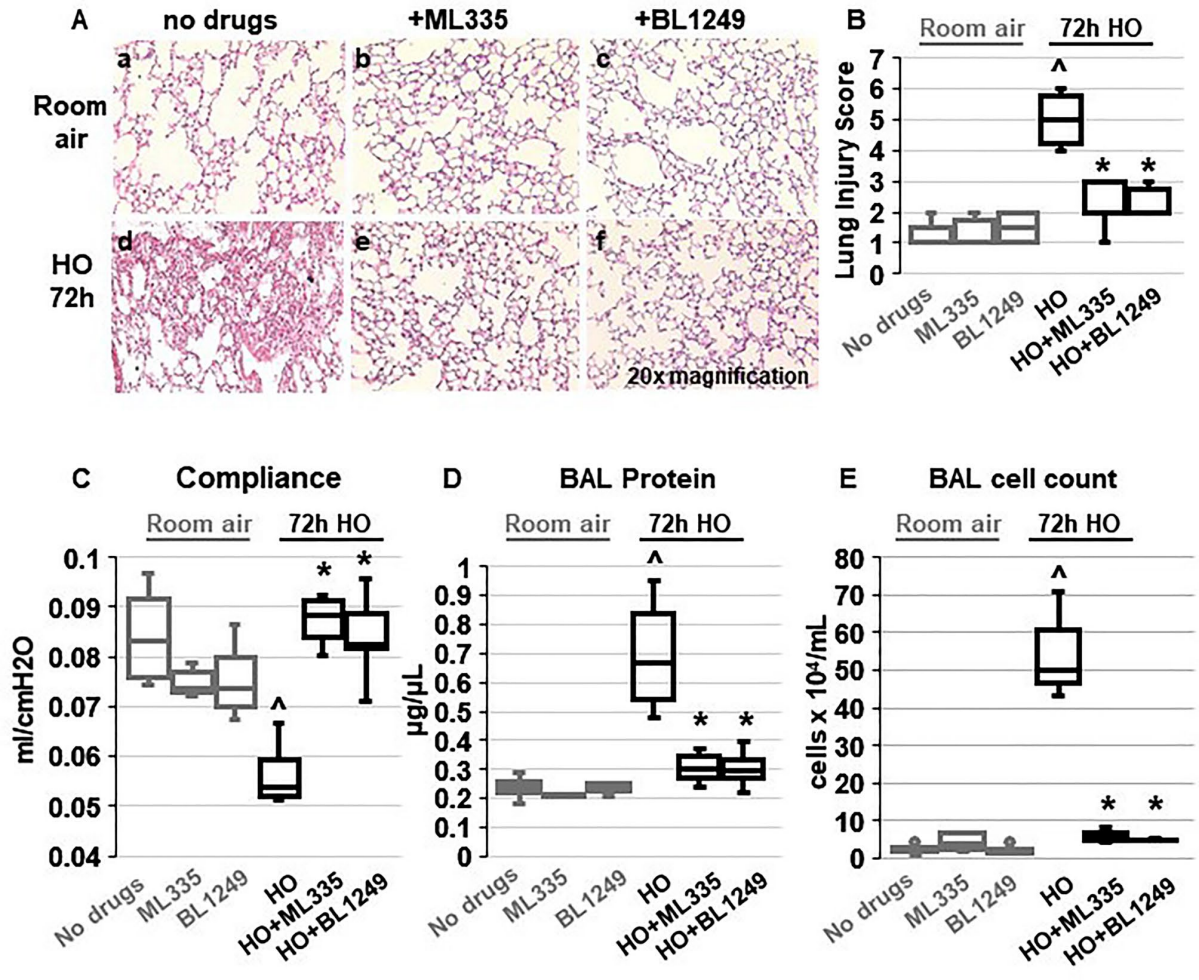
TREK-1 activation with the novel compounds ML335 and BL1249 protects form HO-induced lung injury: (**A**) Representative images of H&E-stained lung sections of WT mice exposed to either room air (panels a-c) or HO (panels d-f) for 72 h. All mice received once-daily, intra-tracheal (*i.t.*) injections of ML335, BL1249, or a vehicle control (no drugs) via brief endotracheal intubation. HO exposure caused significant lung injury (panel d), which was ameliorated by concomitant treatment with ML335 or BL1249 (panels e, f). (**B**) Summary of cumulative Lung Injury Scores of n = 5 independent experiments. (**C**–**E**) The HO-induced decrease in semi-static lung compliance, and increase in BAL fluid total protein and cell count were counteracted by ML335 and BL1249. Data are represented as Box-Whisker plots with medians, 1st and 3rd quartiles, and max and min values; n = 5–9; ^compared to mice injected with a vehicle control and exposed to room air (no drugs), *compared to HO exposed mice; *p* ≤ 0.05.

Building on our previous findings that HO downregulates TREK-1 expression^42^, we determined whether pharmacological activation of the remainder subset of TREK-1 channels can counteract the injurious effects of HO on mouse lungs. We treated WT mice with once-daily intra-tracheal (*i.t.*) injections of the TREK-1-activating compounds ML335 or BL1249^44,45^, or an equimolar drug vehicle control, for a total of 3 injections over the 72-h HO or room air (RA) exposure period. Histological analysis (Fig. 1A) and blinded Lung Injury Scoring (LIS; Fig. 1B) of H&E-stained mouse lung sections revealed that under RA conditions administration of ML335 and BL1249 had no damaging effect on lung histology. As expected, exposure of mice to HO caused significant inflammatory changes (panel d), as also evidenced by an increase in LIS. Importantly, once-daily *i.t.* injections of ML335 or BL1249 during HO exposure substantially reduced these HO-induced injurious effects (Fig. 1A panels e and f, and B). Similarly, analysis of physiological parameters of lung injury also revealed protective effects of TREK-1 activation in HO-exposed mice, as evidenced by improvements in quasi-static lung compliance (Fig. 1C), and a reduction in BAL fluid protein levels and total cell counts (Fig. 1D, E). These data suggest that pharmacological activation of TREK-1 channels can counteract HALI in an experimental mouse model.

### TREK-1 activation decreases inflammatory cytokine concentrations in the BAL fluid of HO exposed mice

**Figure 2:**
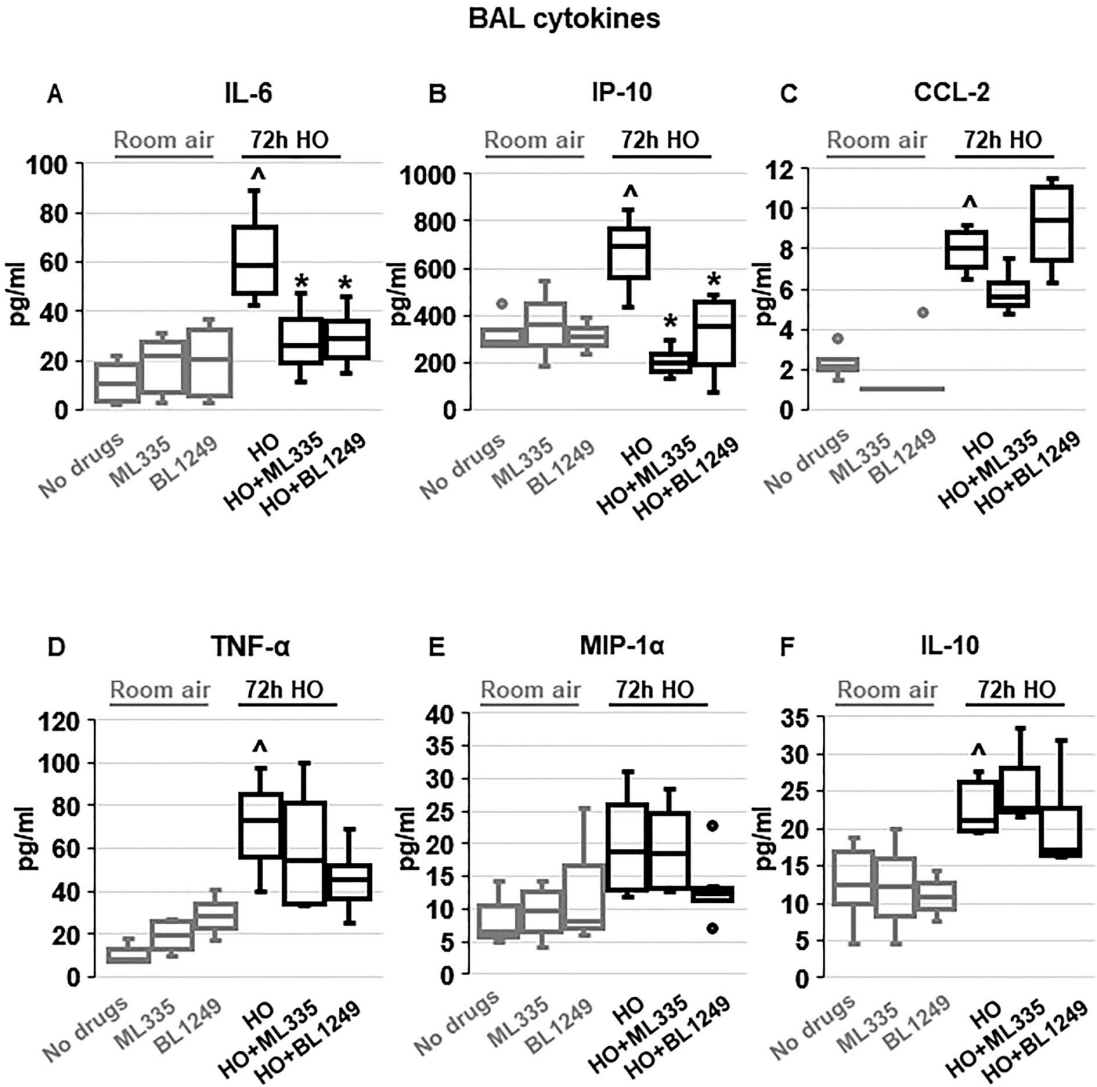
Effects of ML335 and BL1249 on BAL fluid cytokine concentrations (**A**–**F**): HO exposure increased IL-6, IP-10, CCL-2, TNF-α, and IL-10 concentrations, but not MIP-1α. Once-daily *i.t*. treatment with ML335 or BL1249 decreased HO-induced IL-6 and IP-10 levels, but not CCL-2, TNF-α or IL-10. Data are represented as Box-Whisker plots with medians, 1st and 3rd quartiles, and max and min values; n = 5–9; ^compared to mice exposed to room air and treated with a vehicle control (no drugs), *compared to HO exposed mice; *p* ≤ 0.05.

To investigate whether the TREK-1-mediated improvements in histological and physiological lung injury parameters are associated with a reduction in inflammatory cytokine concentrations in HO exposed mice, we measured IL-6, IP-10, CCL-2, TNF-α, MIP-1α and IL-10 concentrations in BAL fluid (Fig. 2). We found that under room air conditions once-daily *i.t.* injections of ML335 or BL1249 had no effect on baseline cytokine secretion. Exposure of mice to 72 h HO resulted in an increase in IL-6, IP-10, CCL-2, TNF-α and IL-10 concentrations in the BAL fluid. Importantly, once-daily *i.t.* injections with ML335 or BL1249 during the 72 h of HO exposure decreased HO-induced IL-6 and IP-10 levels in the BAL fluid, but not CCL-2, TNF-α or IL-10. MIP-1α concentrations were neither affected by HO exposure nor treatment of mice with the TREK-1 activating compounds.

### TREK-1 activity regulates inflammatory cytokine secretion from primary mouse AT2 cells

**Figure 3:**
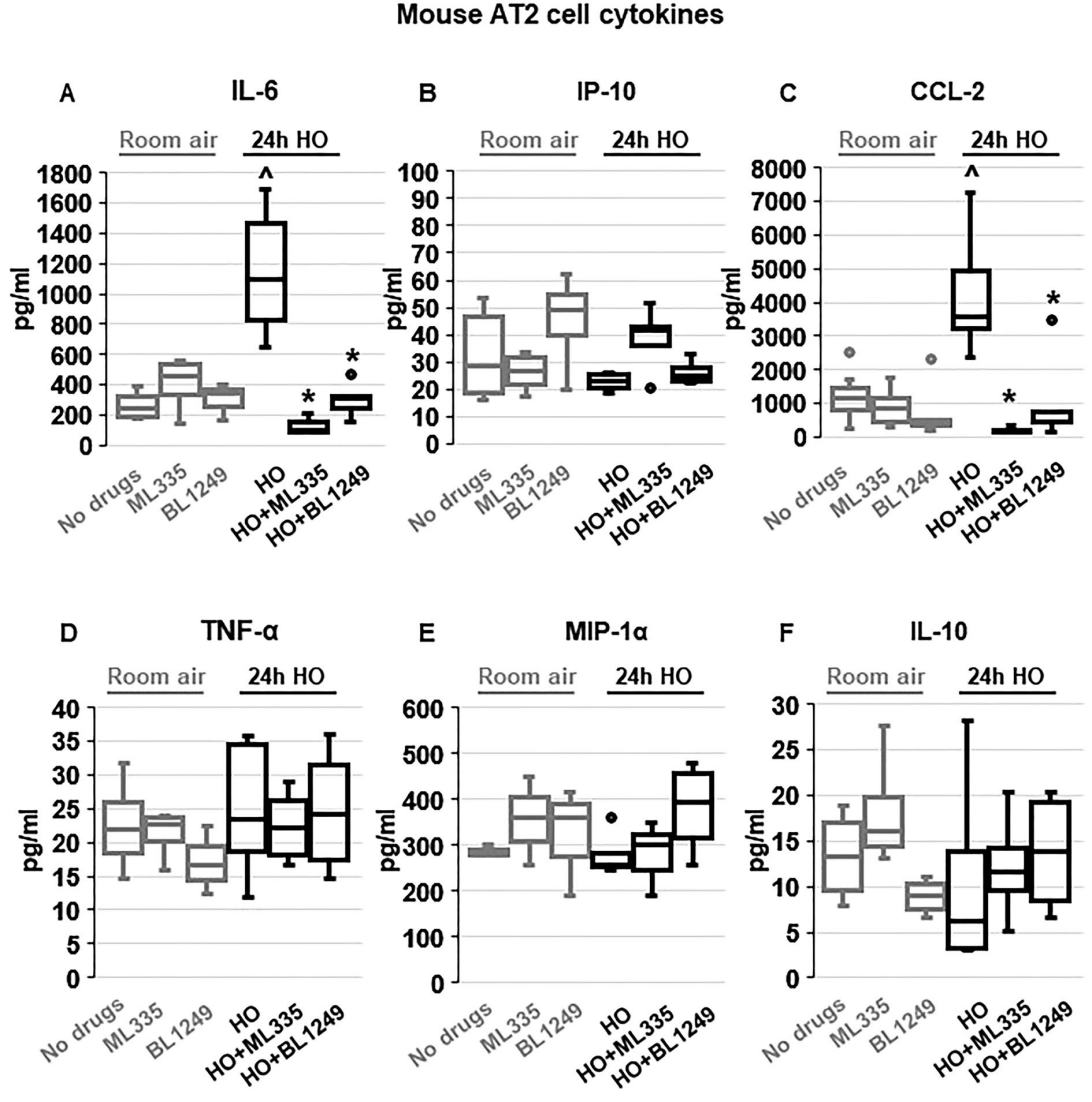
TREK-1 activation with ML335 and BL1249 regulates cytokine secretion from primary mouse AT2 cells: HO exposure increased IL-6 and CCL-2 secretion, which were inhibited by concomitant treatment with ML335 or BL1249 (**A**, **C**). In contrast, IP-10, TNF-α, MIP-1α and IL-10 levels were not affected by TREK-1 activation in room air- or HO-exposed AT2 cells (**B**,**D**,**E**,**F**). Data are represented as Box-Whisker plots with medians, 1st and 3rd quartiles, and max and min values; n = 5–9; ^compared to cells treated with a vehicle control and exposed to room air (no drugs), *compared to HO exposed cells; *p* ≤ 0.05.

To evaluate whether the protective effects of TREK-1 activation observed in vivo were due to TREK-1-mediated attenuation of inflammatory cytokine secretion from alveolar epithelial cells, we exposed freshly-isolated mouse AT2 cells to HO or RA in the presence or absence of ML335 or BL1249, and quantified inflammatory cytokine secretion in culture supernatants (Fig. 3). We chose the shorter (24-h) HO exposure period (compared to 72 h in vivo) for freshly isolated AT2 cells, since in this cell type 72 h of HO exposure resulted in > 60% AT2 cell death (data not shown). Importantly, real-time PCR experiments and immunofluorescence (IF) microscopy imaging confirmed HO-induced TREK-1 downregulation after 24 h in this cell-type (Supplementary Fig. 1A,B). Similar to our findings in the BAL fluid, HO exposure increased secretion of IL-6 and CCL-2 from freshly isolated mouse AT2 cells, and this effect was counteracted by concomitant treatment of cells with the TREK-1 activators ML335 or BL1249. Furthermore, HO exposure did not induce MIP-1α secretion from AT2 cells, similar to our findings in the BAL fluid. In contrast to our findings in the BAL fluid, HO exposure did not induce IP-10, TNF-α or IL-10 secretion from primary AT2 cells, and treatment with ML335 or BL1249 had no effect on the secretion of these cytokines at baseline or after HO exposure (Fig. 3).

### TREK-1 activity regulates inflammatory cytokine secretion from primary human alveolar epithelial cells (HAECs)

**Figure 4:**
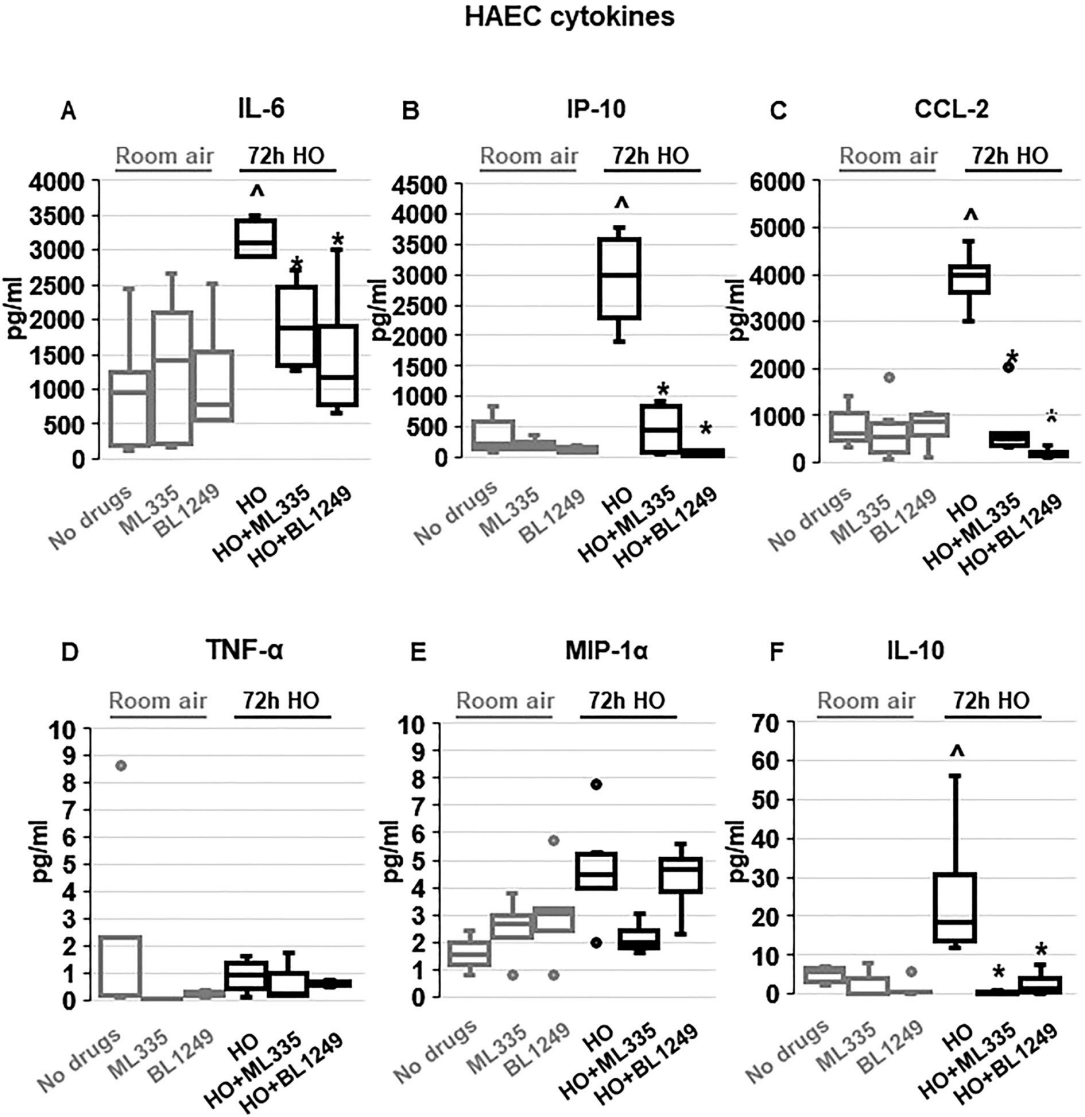
TREK-1 activation with ML335 and BL1249 regulates cytokine secretion from primary human alveolar epithelial cells (HAEC): HO exposure increased secretion of IL-6, IP-10, CCL-2 and IL-10, and this effect was counteracted by ML335 or BL1249 (**A**,**B**,**C**,**F**). In contrast, TNF-α and MIP-1α levels were not affected by TREK-1 activation in room air- or HO-exposed HAECs (**D**,**E**). Data are represented as Box-Whisker plots with medians, 1st and 3rd quartiles, and max and min values; n = 4–8; ^compared to cells treated with a vehicle control and exposed to room air (no drugs), *compared to HO exposed cells; *p* ≤ 0.05.

To determine whether the TREK-1-mediated protective effects observed in mice and mouse AT2 cells can be reproduced in primary human alveolar epithelial cells (HAEC), we exposed HAEC to 72 h HO in the presence or absence of the TREK-1 activators ML335 or BL1249 (Fig. 4). Initial dose–response experiments revealed that BL1249 and ML335 are not cytotoxic at the doses used in this study (Supplementary Fig. 2). In contrast to primary mouse AT2 cells, viability of HAECs after 72 h HO exposure remained > 70% (data not shown), and this exposure period closely mimicked our in vivo model. Similar to our findings in primary mouse AT2 cells, HO exposure increased secretion of IL-6 and CCL-2 from HAECs, but did not induce TNF-α or MIP-1α secretion. Of note, overall concentrations of TNF-α and MIP-1α were quite low in these cells. In contrast to primary mouse AT2 cells but similar to BAL fluid, HO also increased secretion of IP-10 and IL-10 from HAECs. Importantly, treatment of HAECs with the TREK-1 activators ML335 or BL1249 inhibited the HO-induced secretion of IL-6, IP-10, CCL-2, and IL-10.

Altogether, these findings suggest that TREK-1 activation regulates HO-induced inflammatory cytokine secretion both in vivo and in vitro, but differences can be observed between overall cytokine concentrations in BAL fluid and cultured primary epithelial cells.

### ML335 and BL1249 activate TREK-1 currents in primary AT2 cells

**Figure 5:**
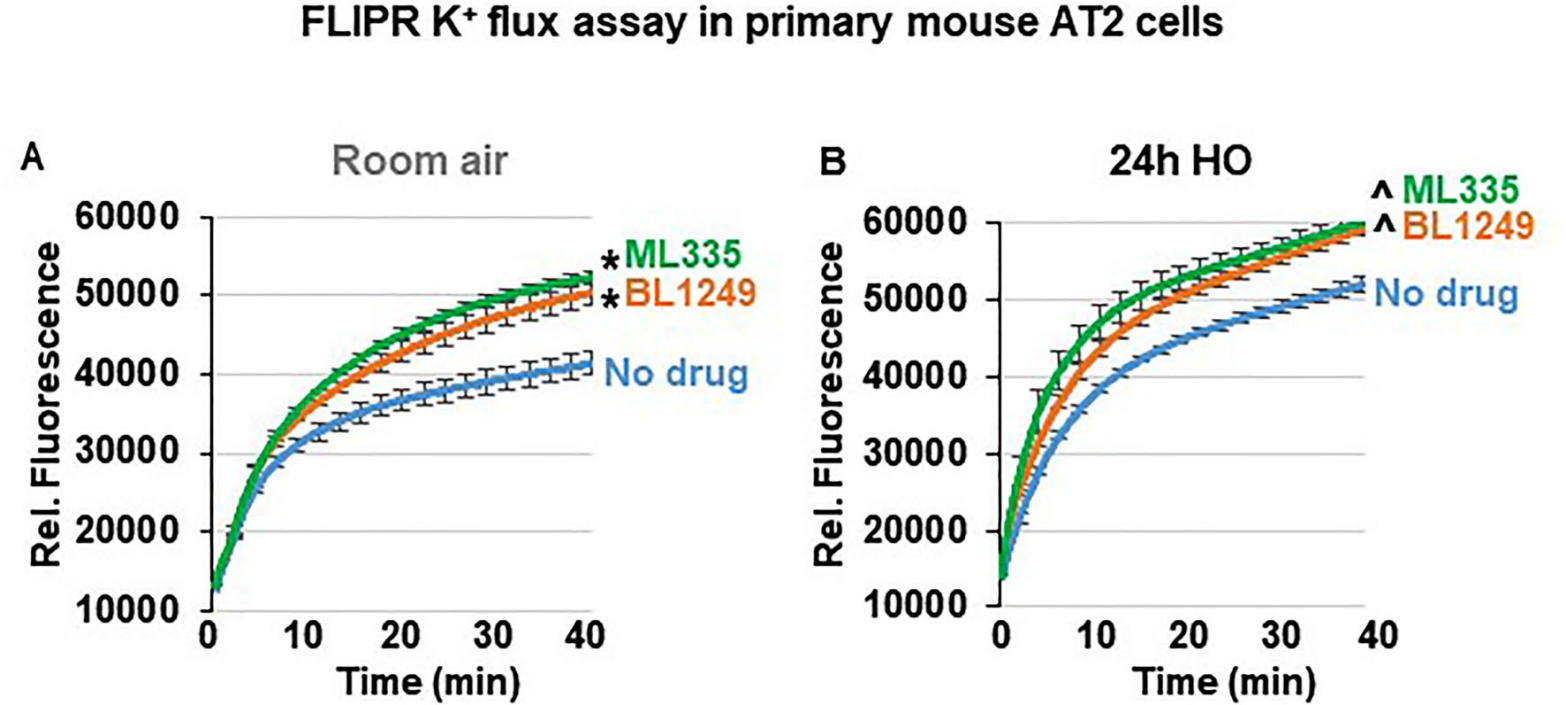
ML335 and BL1249 activate TREK-1 currents in primary mouse AT2 cells: Summary of n = 4–5 independent FLIPR curves (means ± SEM) showing that the TREK-1 activating compounds ML335 or BL1249 induce TREK-1 specific K^+^ currents under both room air and HO conditions (**A**,**B**). In both room air and HO-treated cells, baseline background K^+^ currents were observed (No drug). *compared to vehicle control (No drug) at room air, ^compared to vehicle control (No drug) after HO exposure; *p* ≤ 0.05, n = 4–5, individual experiments were run in triplicates.

Although the specificity of ML335 and BL1249 for TREK-1 channels has previously been validated in heterologous expression systems^44,46,47^, the effectiveness of these compounds has never been demonstrated in lung cells. To confirm that both compounds activate TREK-1 currents in a physiologically relevant system and cell type, we used fluorescence-based, K^+^-sensitive FLIPR assays to demonstrate the effects of ML335 and BL1249 on K^+^ currents in primary mouse AT2 cells (Fig. 5). FLIPR assays exploit the permeability of thallium (Tl^+^) for open K^+^ channels^48^. After loading AT2 cells with the fluorescent dye, the addition of extracellular Tl^+^ creates a concentration gradient for Tl^+^ to enter the cells. The resultant increase in relative fluorescence is proportional to the open probability of plasma membrane K^+^ channels, and thus represents a measure of the functional activity of K^+^ channels. Therefore, under unstimulated conditions (no drugs), the Tl^+^-induced fluorescence represents the sum of background K^+^ currents, while after ML335 or BL1249 treatment an increase in fluorescence represents activation of TREK-1-specific K^+^ currents (Fig. 5). Our data show that under RA conditions both compounds, ML335 and BL1249, activate TREK-1-specific K^+^ currents (Fig. 5A). Importantly, these effects were maintained after 24 h of HO exposure (Fig. 5B).

### Activation of TREK-1 channels hyperpolarizes the plasma membrane potential (Em) of primary AT2 cells

**Figure 6:**
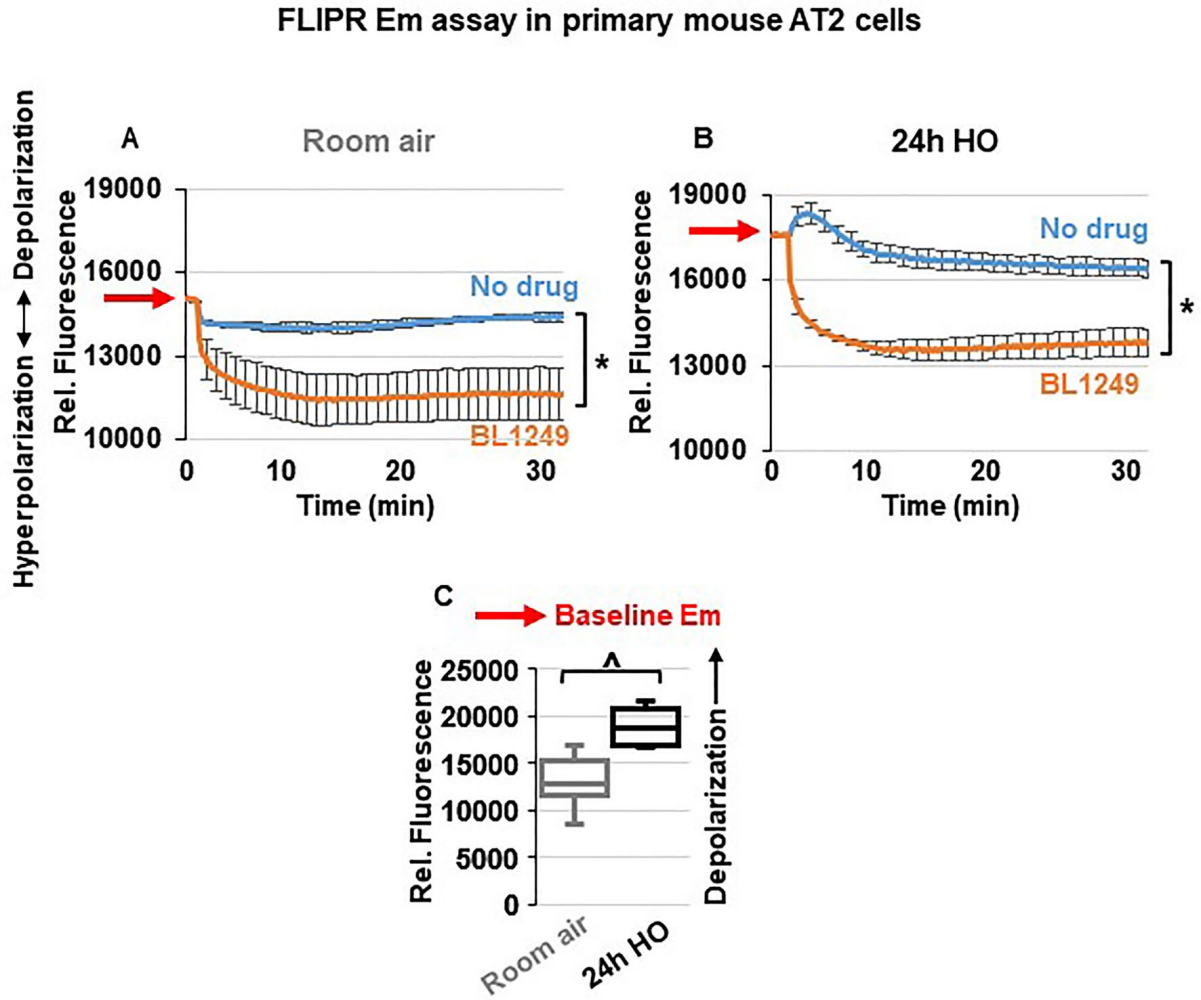
TREK-1 activation causes plasma membrane potential (Em) hyperpolarization: Representative curves of Em-sensitive FLIPR assays showing that TREK-1 activation with BL1249 causes Em hyperpolarization in primary mouse AT2 cells under both room air and HO conditions (**A**,**B**), as indicated by a decrease in fluorescence values. Red arrows on the Y-axis indicate relative fluorescence values reflective of the baseline Em value in room air and HO exposed AT2 cells, demonstrating that HO exposure itself causes Em depolarization (higher baseline fluorescence value in B than A; *BL1249 compared to no drug/vehicle control, *p* ≤ 0.05). (**C**) Summary of baseline Em values of RA- vs. HO-exposed AT2 cells averaging n = 6 independent experiments for each condition. Data are represented as Box-Whisker plots with medians, 1st and 3rd quartiles, and max and min values; ^compared to room air exposed AT2 cells; *p* ≤ 0.05, individual experiments were run in triplicates.

To determine whether the protective effects of ML335 and BL1249 are mediated by TREK-1-induced alterations in the Em, we performed Em-sensitive FLIPR assays on primary mouse AT2 cells under RA and HO conditions (Fig. 6). Once cells are loaded with the Em-sensitive fluorescent dye, a decrease in relative fluorescence represents Em hyperpolarization, whereas an increase in fluorescence represents Em depolarization. Since both TREK-1 activating compounds, ML335 and BL1249, had identical effects (i) in our in vivo model, (ii) on inflammatory cytokine secretion, and (iii) on TREK-1 current activation, we used only BL1249 for this part of the study. We found that under room air conditions, activation of TREK-1 channels with BL1249 results in Em hyperpolarization (= a decrease in fluorescence; Fig. 6A) of primary AT2 cells. Similar to the observed activation of TREK-1 currents with BL1249 (Fig. 5), the BL1249-induced Em hyperpolarization also persisted after HO exposure (Fig. 6B). Importantly, these studies also revealed that HO itself causes Em depolarization when compared to cells kept at RA, as evidenced by a higher baseline fluorescence value in HO-exposed cells (see RED arrows on the Y-axis → in Fig. 6A, B and summarized in C).

### TREK-1 activation decreases intracellular Ca^2+^ (iCa) levels during HO exposure

**Figure 7:**
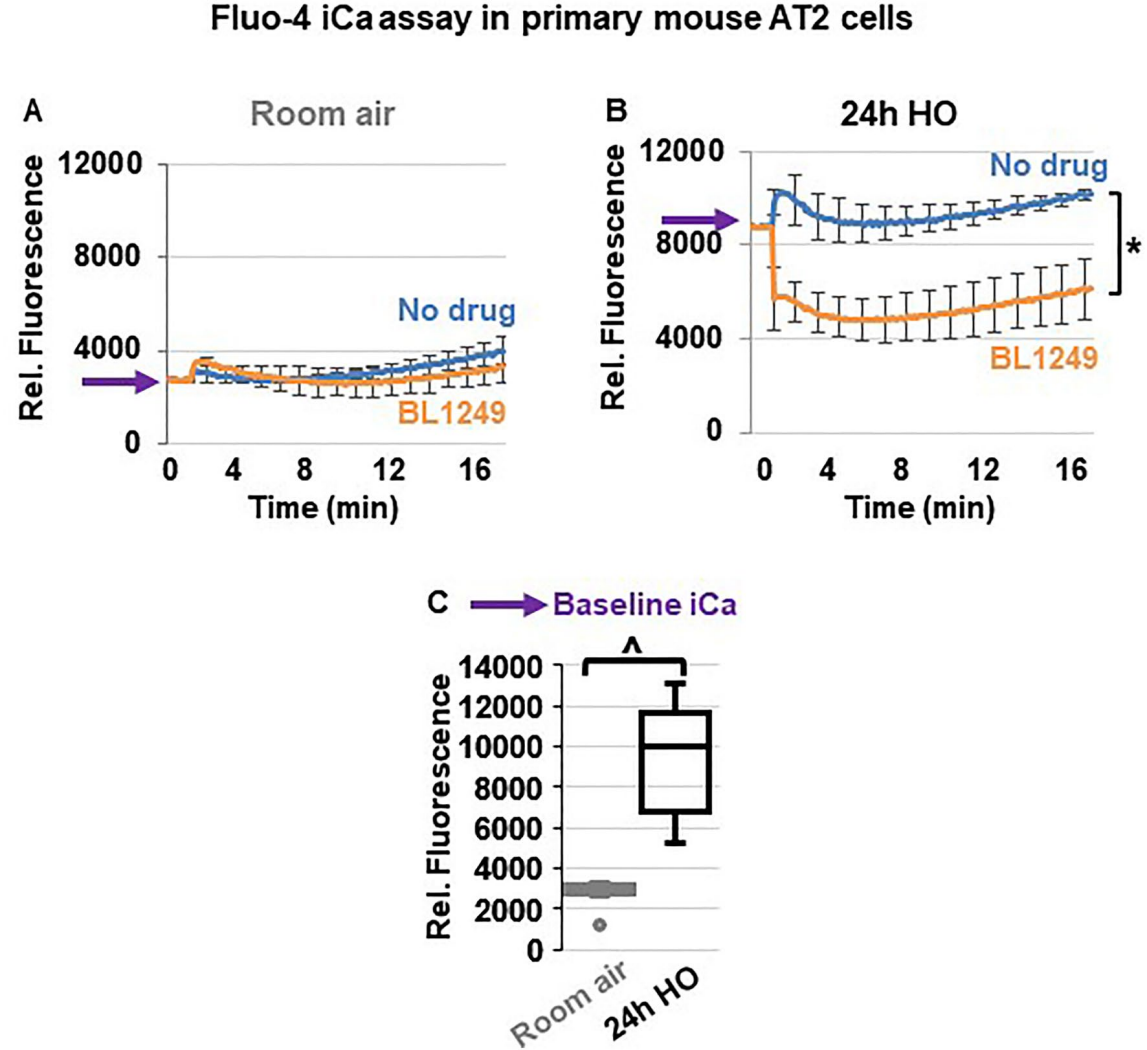
TREK-1 activation decreases intracellular Ca^2+^ (iCa) concentrations in HO-exposed primary mouse AT2 cells: (**A**,**B**) Representative curves of Ca^2+^-sensitive Fluo-4 assays showing that HO-exposed AT2 cells contain higher iCa concentrations than RA-exposed cells, as indicated by an increase in fluorescence values (purple arrows on Y-axes). TREK-1 activation with BL1249 has no effect on iCa concentrations in RA-exposed cells, but decreases iCa levels in HO-exposed cells (*BL1249 compared to no drug/vehicle control, *p* ≤ 0.05). A summary of n = 6 independent experiments is shown in **C**; data are represented as Box-Whisker plots with medians, 1st and 3rd quartiles, and max and min values; ^compared to room air exposed AT2 cells; *p* ≤ 0.05, individual experiments were run in triplicates.

Since inflammatory cytokine secretion is commonly associated with an increase in iCa concentrations, we used Fluo-4 assays to determine the effects of TREK-1 activation on iCa levels in primary mouse AT2 cells (Fig. 7). Importantly, following 24 h of HO exposure, AT2 cells contained higher iCa concentrations than cells kept at RA (PURPLE arrows on the Y-axis → in Fig. 7A,B and summarized in C). In cells kept at RA, activation of TREK-1 channels and Em hyperpolarization with BL1249 had no effect on iCa levels, likely due to the already low iCa levels in resting cells (Fig. 7A). In HO-exposed cells, on the other hand, TREK-1 activation with BL1249 decreased the HO-induced elevation in iCa levels (Fig. 7B).

Altogether, these findings highlight the TREK-1 activating effects and resultant Em hyperpolarization caused by ML335 and BL1249 in primary alveolar epithelial cells, and demonstrate that these effects persist under HO conditions, making TREK-1 activation a feasible approach to modulate the Em and iCa concentrations during HO exposure.

### Regulation of inflammatory cytokine secretion by voltage-gated Ca^2+^ (Ca_V_) channels

**Figure 8:**
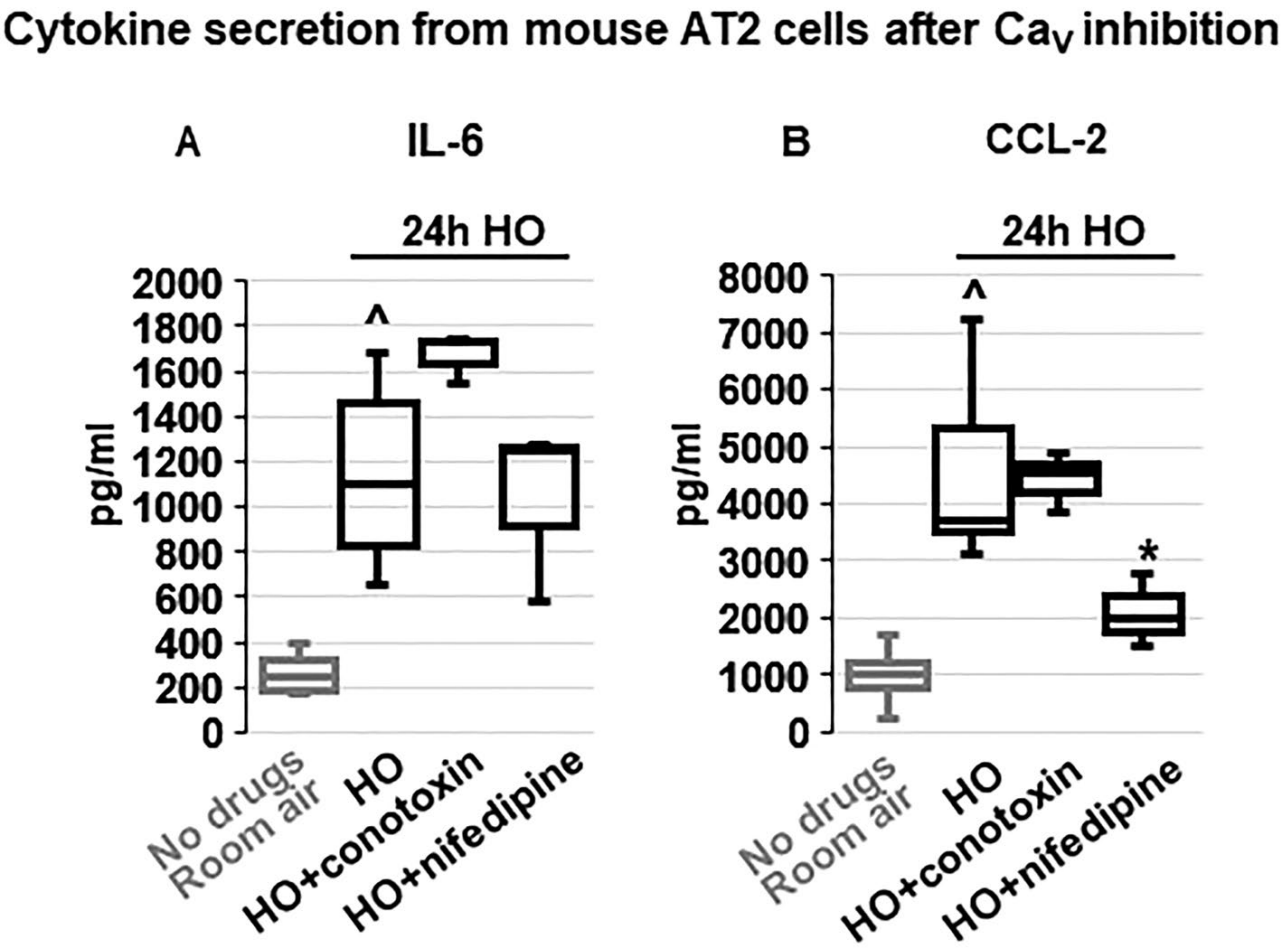
Effects of voltage-gated Ca^2+^ channel (Ca_V_) inhibition on cytokine secretion from primary mouse AT2 cells: HO exposure increased IL-6 (**A**) and CCL-2 (**B**) secretion compared to RA-treated cells. Inhibition of N- and P/Q-type Ca_V_ channels with ω-conotoxin MVIIC or L-type Ca_V_ channels with nifedipine revealed that IL-6 secretion occurred independently of Ca_V_ channel activity, whereas CCL-2 secretion was dependent on L-type Ca_V_ channels (inhibited by nifedipine) but not N- and P/Q-type channels (lack of ω-conotoxin MVIIC effect). Data are represented as Box-Whisker plots with medians, 1st and 3rd quartiles, and max and min values; n = 3–6; ^compared to cells treated with a vehicle control and exposed to room air (No drugs), *compared to HO exposed cells; *p* ≤ 0.05.

Em depolarization, as observed with HO exposure and counteracted by TREK-1 activation, results in opening of Ca_V_ channels in many cell types, and the resultant increase in iCa concentrations is commonly a trigger for downstream inflammatory cytokine secretion^49,50^. Therefore, we measured HO-induced cytokine secretion from primary mouse AT2 cells after blocking N- and P/Q-type Ca_V_ channels with ω-conotoxin MVIIC^51^, and L-type Ca_V_ channels with nifedipine^52^ (Fig. 8). Since in primary AT2 cells HO exposure predominantly induced secretion of IL-6 and CCL-2 (Fig. 3), we focused on the role of Ca_V_ channels in the secretion of these two cytokines. Interestingly, while HO-induced IL-6 secretion was not dependent on Ca_V_ channel activity, CCL-2 secretion was inhibited by the L-type Ca_V_ channel blocker nifedipine, but not by the N- and P/Q-type Ca_V_ channel blocker ω-conotoxin MVIIC. Secretion of IP-10, TNF-α, MIP-1α and IL-10 from AT2 cells was not affected by ω-conotoxin MVIIC or nifedipine (data not shown).

Altogether, these findings suggest that regulation of inflammatory cytokine secretion via TREK-1-induced Em hyperpolarization and inhibition of Ca_V_ channel activation could explain some, but not all, of the TREK-1 protective effects seen in our in vivo model.

## Discussion

In this study we propose activation of TREK-1 K^+^ channels as a potentially new therapeutic approach against HALI, since currently no targeted interventions exist that translate into improved patient outcomes. Recent in vitro studies suggest that overexpression of certain microRNAs (miR16, miR21-5) may protect cultured AT2 cells against HO-induced apoptosis^53–55^, and multiple biomarker studies have aimed at predicting the risk of HALI in patients^56,57^. In addition, neutralizing therapies against individual cytokines, including IL-6, TNF-α and CCL-2, have yielded variable results at best in improving inflammatory responses^58–61^.

Given these challenges, we are particularly interested in identifying strategies that can regulate multiple inflammatory pathways simultaneously, such as the manipulation of the plasma membrane potential (Em). We previously discovered that HO downregulates TREK-1 K^+^ channel expression in lung tissue and alveolar epithelial cells, which correlates with worsening lung injury and alterations in multiple inflammatory cytokines (IL-6, CCL-2, RANTES, and IL-1β)^29–32,42^. Importantly, this HO-induced decrease in TREK-1 expression leaves a remainder subset of TREK-1 channels suitable for pharmacological activation. Although channels of the K2P family are known for their so-called “leak K^+^ currents” (a constant, slow K^+^ efflux that stabilizes the Em), TREK-1 channels are actually thought to be closed at baseline^34,62^. This idea is supported by our own data in alveolar epithelial cells (Fig. 5) showing that TREK-1 currents can readily be induced by our channel activators ML335 and BL1249^44,46,47^, thus making TREK-1 channels a feasible target for therapeutic activation.

So far, most of the biophysical characterization of TREK-1 channels has occurred under non-physiological conditions in heterologous expression systems^44,63,64^, and little is known about their functions in physiologically-relevant models. Our study is the first to (a) report the safety and efficacy of the novel TREK-1 activating compounds ML335 and BL1249 in an in vivo system, and (b) highlight the protective effects of TREK-1 activation in a lung injury model by measuring clinically relevant parameters. The only other reports suggesting a potentially protective role for TREK-1 activation used models of hypoxic-ischemic brain injury and atrial fibrillation/heart failure^65–67^. Interestingly, effects of single nucleotide polymorphisms (SNPs) in the human TREK-1 gene have been reported in the same two organs, and predict resistance to antidepressant medication^68^, and an increased risk for atrial tachycardias^69^. However, until now a similar protective effect for TREK-1 channels has not been reported in any other organ.

The importance of inflammatory mediators in the development and progression of HALI is well-established^14,70^, including the cytokines reported in this study: IL-6, IP-10, CCL-2, TNF-α, MIP-1α, and IL-10^17,21^. In general, IL-6, IP-10, TNF-α and MIP-1α are known for their proinflammatory properties, while CCL-2 can exert pro-^71,72^ or anti-inflammatory^73,74^ effects, and IL-10 is considered a predominantly anti-inflammatory cytokine^75,76^. More recently it has become increasingly clear that the inflammatory phenotypes observed in various lung injury models are determined by complex interactions between multiple cytokines. For example, despite the well-documented proinflammatory effects of IL-6 and its association with poor outcomes in ARDS patients^77^, IL-6 also induces anti-inflammatory IL-10 secretion as a counter-regulatory response^78^, and a recent study suggests that IL-6 protects mice from LPS- and mechanical ventilation-induced lung injury^79^. In our HALI model we found increased levels of both IL-6 and IL-10 in the BAL fluid of HO-exposed mice. Interestingly, while TREK-1 activation decreased HO-induced BAL fluid IL-6 levels, IL-10 levels remained elevated even after TREK-1 activation, potentially acting synergistically with the protective effects of TREK-1 activation. It is important to note that both lung resident and immune cells contribute to the cytokine levels measured in BAL fluid, and it is quite likely that in our in vivo model the TREK-1 activators affect cytokine secretion from multiple cell types. In this study we focused on epithelial cells since previously we did not find alterations in TNF-α release from TREK-1-deficient alveolar macrophages, and the single cell RNA-seq database LungGENS only reports low levels of TREK-1 postnatally in endothelial cells^42,80^.

Our results report for the first time (1) the expression of functional TREK-1 channels on primary mouse AT2 cells and human alveolar epithelial cells (HAEC), and (2) the effects of Em manipulation via TREK-1 channels on inflammatory cytokine secretion and iCa concentrations in a clinically relevant model of HALI. Since in clinical practice the timing of HO therapy is entirely under the control of the healthcare provider, administration of TREK-1 activators simultaneously with initiation of HO therapy is a clinically feasible approach. Of note, although in animal models the injurious effects of HO on previously healthy lungs have been extensively studied, in humans the exact degree and duration of HO exposure that results in symptomatic and clinically-relevant injury remains a matter of intense discussion^81^.

From studies in macrophages, neutrophils and mast cells, we learned that changes in the Em commonly precede secretory events^82,83^, but the molecular mechanisms regulating inflammatory cytokine secretion from lung resident cells remain incompletely understood. Furthermore, studies in lung endothelial cells, revealed that the resting Em can vary among cell phenotypes. Reported Em values in endothelial cells range from − 30 to − 60 mV^84,85^, and exposure of pulmonary artery endothelial cells to low oxygen concentrations (hypoxia) has been reported to cause Em depolarization^86^. Similar variations in Em depending on the cellular phenotype have also been documented in lung epithelial cells, including rat AT2 cells (− 30 mV)^87,88^, rabbit AT2 cells (− 60 mV)^89^, human bronchial epithelial cells (− 20 to − 45 mV)^90,91^, and nasal epithelial cells (− 15 to − 30 mV)^91,92^. Limited information form human ex vivo studies point towards Em values between − 15 and − 20 mV in bronchial epithelial cells^91,93^. Notably, other studies estimate the resting Em in AT2 cells as low as 0 to − 5 mV^94,95^. Despite these ranges in Em for lung resident cells, it is important to realize that the Em of epithelial and endothelial cells is much lower than the Em of excitable cells such as neurons and cardiomyocytes, in which the Em ranges between − 60 and − 90 mV^96,97^. Since these latter cell types are more hyperpolarized at baseline (i.e. more negative Em values), they require a much stronger depolarization stimulus for a biological response to occur, such as the opening of voltage-gated Ca^2+^ (Ca_V_) channels and subsequent Ca^2+^ influx. In contrast, in the more depolarized epithelial and endothelial cells, a much smaller Em perturbation can reach the threshold for Ca_V_ channel activation, and trigger downstream responses. Conversely, K^+^ efflux, as caused by TREK-1 activation with BL1249 (Fig. 6A), moves the Em away from this critical threshold towards more negative (hyperpolarized) Em values, and can counteract depolarization-induced cell activation processes.

HO-mediated depolarization events have been reported in mitochondrial membranes of pulmonary endothelial cells^98^, but our study is the first to show HO-induced Em depolarization in primary epithelial cells. Interestingly, in carotid body cells hypoxia, not hyperoxia, causes Em depolarization and increases iCa^2+^ concentrations, while HO inhibits both of these processes^99,100^. In contrast to these studies, we demonstrate that primary epithelial cells respond to HO exposure by increasing iCa^2+^ levels (Fig. 7), and we propose that this response is mediated by HO-induced Em depolarization that can be counteracted by TREK-1 activation (Figs. 6, 7).

Interestingly, although it is well-known that both extracellular Ca^2+^ influx and Ca^2+^ release from intracellular stores can increase iCa^2+^ levels, we found that in primary mouse AT2 cells only secretion of CCL-2, but not IL-6, IP-10, TNF-α, or MIP-1α, was dependent on Ca^2+^ influx via Ca_V_ channels (Fig. 8). The lack of effect of ω-conotoxin MVIIC on cytokine secretion suggests that Ca^2+^ influx via N-, and P/Q-type Ca_V_ channels is unlikely to contribute to these processes. In addition to the novelty and importance of our data, these findings also indicate that Ca^2+^ release from intracellular stores is likely to be involved in the observed secretory processes.

Although upregulation of CCL-2 in bronchial and alveolar epithelial cells under inflammatory conditions is well-documented^101–103^, it remains a matter of intense discussion whether CCL-2 secretion in the lung is a Ca^2+^-dependent process, and may ultimately depend on the specific cell type and inflammatory environment. In both immortalized and primary lung epithelial cells, inhibition of Ca^2+^ sensing, Ca^2+^ influx, and iCa^2+^ release all prevent CCL-2 secretion, and in some instances also IL-6 release^104,105^. Conversely, it is known that in immune cells CCL-2 itself can increase iCa^2+^ concentrations^106^, demonstrating the complex interactions underlying CCL-2 secretion. One study showed that the chemotactic function of CCL-2 can occur in the absence of any changes in iCa^107^, and in an LPS-induced lung injury model inhibition of cellular Ca^2+^ sensing receptors (CaSR) decreased IL-6 and TNF-α, but not CCL-2, concentrations in the serum and BAL fluid^104^.

Since in our model inhibition of Ca_V_ channels decreased CCL-2 secretion but no other measured cytokines, we should consider the possibility that TREK-1-induced changes in Em could be directly sensed by a voltage-sensitive protein at the plasma membrane level. For this to occur, such a protein would need to contain one or more transmembrane segments with free charges that can induce a so-called “gating current” following an alteration in Em. Although membrane-bound voltage sensors are well-characterized in the brain and heart^108,109^, in the lung this important topic has yet to be explored.

We previously reported an important role for TREK-1 in HALI using a TREK-1-deficient mouse model^42^, which revealed a similar injurious phenotype as can be obtained with HO-induced TREK-1 downregulation^42^. In this study, we now shed some light on how TREK-1 may regulate downstream signaling cascades during HO exposure. Based on the current and our previous studies, we propose that the primary mechanism underlying the HO-mediated effects on TREK-1 signaling consists in a decrease in TREK gene and protein expression levels, rather than potential HO-mediated post-translational modifications of the TREK-1 protein structure. Of note, in HEK293 cells, posttranslational TREK-1 phosphorylation has been reported, and resulted in TREK-1 inhibition^110^. However, even if such changes occurred in the lung, they do not seem to interfere with the activation effects of BL1249 and ML335 on TREK-1 channels. Since BL1249 and ML335 are designed to bind and functionally activate wildtype TREK-1 channels, substantial HO-induced structural/posttranslational changes to the TREK-1 structure are unlikely the cause for our reported outcomes. In fact, one of the key findings of this study is that BL1249 and ML335 can activate TREK-1 channels and ameliorate injury despite any HO-induced changes in the intra- and extracellular cellular environments. Notably, we previously reported TREK-1 expression in both AT1 and AT2 cells from mouse lung slices, as well as mouse alveolar macrophages (AMs), but saw only weak TREK-1 staining in the mouse lung endothelium. Interestingly, in that study we also found that LPS-induced TNF-α release from mouse AMs appears to occur independently of TREK-1^31^, suggesting that epithelial TREK-1 channels are the primary target for BL1249 and ML335 in our HALI model.

In conclusion, we report for the first time the functional expression of TREK- 1K^+^ channels on primary alveolar epithelial cells. We show that pharmacological activation of TREK-1 channels during HO exposure is a novel and clinically feasible approach to protect against HALI by reducing inflammatory cell recruitment and barrier dysfunction in the lungs, which may at least in part be mediated by inhibition of inflammatory cytokine secretion. However, additional studies are required to identify other potential effector mechanisms contributing to TREK-1-mediated protection, which should include ROS production, cell death pathways, and inflammasome activation.

## Materials and methods

### Mice

C57bl/6 wild-type (WT) mice aged 9–12 weeks were obtained from Jackson Laboratories (www.jax.org). Mice were housed in same-sex groups of up to 5 mice per cage and provided with food and water ad libitum. For experimental purposes, mice were age- and gender-matched as closely as possible.

### Mouse hyperoxia (HO) exposure

Using a rodent HO chamber and a 5-L oxygen concentrator (DeVilbiss Healthcare, #525DS), we exposed mice to HO (F_i_O_2_ = 0.8–0.9 inside the chamber) for 72 h in their native cages with free access to food and water. Temperature, humidity and oxygen concentrations were monitored continuously using commercially available sensors (AcuRite 00325A1 for temperature and humidity; Hudson-RCI5800 for oxygen concentrations). During HO exposure, mice lost less than 10% of weight and appeared overall healthy. No deaths were observed. Control mice were exposed to room air (RA) for the same time period in their native cages.

### TREK-1 activating compounds

We used two novel TREK-1 activating compounds, ML335 and BL1249. ML335 has been synthesized and validated by our collaborator Dr. Minor at UCSF^44^, who provided this compound to us as gift. BL1249 has most recently become commercially available (Tocris)^45^. Stock solutions for ML335 (100 mM) and BL1249 (100 mM) were prepared in DMSO. For in vivo experiments, we used a final concentration of 100 μM ML335 and 200 μM BL1249 in sterile PBS. For in vitro experiments in primary cells, we used a final concentration of 100 μM (60 μg/kg) ML335 and 10 μM (100 μg/kg) BL1249 suspended in culture media. Vehicle controls for all experiments contained equimolar amounts of the DMSO.

### Intra-tracheal injections

During the 72 h of RA or HO exposure, mice were injected once-daily intratracheally (*i.t.*) via brief endotracheal intubation with either 40μL of the TREK-1 activating compounds ML335, BL1249, or a vehicle control in sterile PBS. Briefly, for *i.t.* injections, mice underwent brief inhaled isofluorane (2–5%) anesthesia until they lost consciousness, and were then suspended by their incisors on a 3.0 silk suture mounted on a 45 degree-angled stand. The tongue was gently extracted from the mouth and moved to the side using blunt forceps in order to visualize the vocal cords. Using fiberoptic guidance, a 20-gauge angiocatheter was passed through the vocal cords into the subglottic area, and 40 μL of drug or vehicle control were injected with a micropipettor. Mice were then placed back into their native cages and allowed to recover under a warming lamp until fully awake. No perianesthetic deaths were associated with this procedure.

### Quasi-static lung compliance measurements

Following RA or HO exposure, a tracheostomy was performed using an 18-gauge steel catheter under general ketamine/xylazine anesthesia (intraperitoneal, 10 mg/kg ketamine; 20 mg/kg xylazine). Quasi-static lung compliance was measured using the Flexivent system (SQIREC). Pressure–volume curves (P–V) were recorded, and each set of P–V curves was preceded by an inflation maneuver to total lung capacity to insure equal standard lung volumes for each experiment. Quasi-static lung compliance was calculated by fitting data derived from the P–V curves to the Salazar-Knowles equation as previously described^111^. Rectal temperatures were maintained in physiologic range using a heat lamp. All experiments were terminal.

### Broncho-alveolar lavage (BAL) fluid collection and lung histology

Following Flexivent measurements, BAL fluid was collected from all mice using a 1 ml syringe attached to the tracheostomy catheter. Two wash-outs were performed with 1 ml PBS/0.6 mM EDTA for BAL protein and cell count determination, and 1 ml PBS/0.5% BSA for cytokine assays. All samples were immediately placed on ice. Total BAL protein concentrations were measured using the Bradford assay (BioRad), and total BAL cell counts were performed using a Diff-Quick stain (Fisher Scientific). Thereafter, lung tissue was harvested and processed for histological examination. Briefly, the lungs were gently retrograde perfused via the right ventricle with 10 ml ice-cold PBS to remove red blood cells. Lung tissue was then removed *en bloc* and immediately perfused and fixed in 4% formalin. Paraffin-embedded sections were cut into 4 µm thick tissues slices using a Microtome, and H&E-stained for histological analysis. Lung Injury Scores (LIS) were determined by an investigator blinded to the experimental conditions on H&E-stained lung sections as previously described, using the following 3 criteria: (1) interstitial and alveolar edema, (2) cellular infiltrate, and 3) parenchymal and perivascular hemorrhage. Each criterion was assigned a score between 0–3, with “0” representing no injury, “1” representing mild injury, “2” representing moderate injury, and “3” representing severe injury. Five randomly assigned high power fields per slide were scored under 40 × magnification on a Motic AE20/21 inverted microscope, and scores were averaged for each criterion. Using the sum of these averages, a composite histological LIS was calculated for each experimental group.

### Primary mouse and human alveolar epithelial cells

Primary mouse alveolar type-2 cells (AT2) cells were freshly isolated as previously described^112^. We obtained in average 3–5 × 10^6^ AT2 cells per mouse lung with > 90% purity as assessed by immunostaining for pro-SPC. All experiments were performed in accordance with the guidelines of the Institutional Animal Care and Use Committee at the University of California Los Angeles. Freshly isolated AT2 cells were seeded to 70–80% confluence at a density 3.5 × 10^6^ cells per well in 6-well tissue culture plates coated with fibronectin. Cells were maintained in DMEM cell culture medium containing 10% FBS, 4 mM glutamine, 1% penicillin/streptomycin, and 0.25 µM amphotericin B. All experimental interventions were started on day 2 after AT2 cell isolation.

Primary Human Alveolar Epithelial Cells (HAEC) were purchased from ScienCell (#3200), cultured according to the company’s instructions, and used at a passage numbers < P5. Since these cell suspensions are directly isolated from donated human lung tissue, they contain mixed populations of AT1 and AT2 cells.

### HO exposure of cells

HO exposure of cells was performed using a cell culture-compatible HO chamber. HAEC were exposed to 72 h of HO to mimic our in vivo HO protocol. Since in freshly isolated mouse AT2 cells we observed substantial cell death after 72 h of HO exposure (F_i_O_2_ 0.8–0.9), we limited HO exposure to 24 h for these cells. Controls for each cell type were cultured at room air for the respective time intervals. During the HO or RA exposure period, cell suspensions were treated with a one-time dose of the TREK-1 activating compounds ML335 (100 μM) or BL1249 (10 μM), or an equimolar DMSO vehicle control. Under all experimental conditions cell viability remained greater than 75% as determined by Trypan Blue staining. To assure that BL1249 and ML335 were not cytotoxic at the doses used, we performed dose–response experiments using two cell viability assays, CCK-8 (APExBIO) and XTT (Biotium).

### TREK-1 gene and protein expression

We used real-time PCR and IF microscopy to confirm HO-induced TREK-1 downregulation after 24 h in freshly isolated mouse AT2 cells. Briefly, for PCR experiments total RNA was isolated using a Qiagen RNeasy Mini Kit (Hilden, Germany), 1 μg RNA was reverse transcribed with a High Capacity cDNA Reverse Transcription kit (Applied Biosystems), and amplified by semi-quantitative real-time PCR (TaqMan) with primers specific for TREK-1 (KCNK2; Applied Biosystems). For IF microscopy, mouse AT2 cells were fixed with 4% paraformaldehyde and then incubated with an anti-TREK-1 primary antibody (Alomone, 1:200) at 4 °C overnight, followed by probing with a species-specific secondary antibody (1:1000; Abcam) for one hour at room temperature. Nuclei were counterstained with Fluoro Gel II mounting medium containing DAPI (EMS). All images were recorded using Zen 2009 Light Edition software version 5.5 (Zeiss; https://www.zeiss.com/microscopy/us/products/microscope-software/zen-lite.html).

### Cytokine measurements by ELISA

Cytokine concentrations were quantified in BAL fluid and cell culture supernatants after centrifugation at 8000 rpm for 5 min. Briefly, 100 μL of sample was loaded into 96-well ELISA plate, and analyzed following the manufacturer’s instructions. All samples were run in triplicates and values are displayed in pg/mL. Species-specific ELISA kits were purchased from the following vendors: IL-6 (BD Biosciences), IP-10 (mouse: R&D Systems; human: BD Biosciences), CCL-2 (BD Biosciences), TNF-α (BD Biosciences), MIP-1α (R&D Systems), IL-10 (R&D Systems).

### FLIPR and Fluo-4 assays for K^+^ flux, plasma membrane potential (Em), and intracellular Ca^2+^ (iCa) measurements

K^+^ channel activity and Em measurements were performed using commercially available FLIPR assays (Molecular Devices, #R8222 and #R8126, respectively), and Fluo-4 assays (Invitrogen, #F36206) for iCa measurements. All three assays were performed following the manufacturer’s instructions. Briefly, for all assays 30,000 cells/well were seeded into dark-walled, clear-bottom 96-well plates (Grenier Bio-One, #655090), and cultured in growth medium overnight. The next day, cells were washed once and incubated at 37 °C with the respective loading dye for 60 min for K^+^ channel activity assays, and 30 min for Em and Fluo-4 assays. In all assays, fluorescence traces were recorded for 1 min to reach a stable baseline before the addition of any drugs. All plates were analyzed using a BioTek Synergy-2 fluorescence plate reader. Data points were collected and integrated every 7 s. To determine whether an increase in iCa concentrations was due to Ca^2+^ influx via voltage-gated Ca^2+^ (Ca_V_) channels, we blocked N- and P/Q-type Ca_V_ channels with ω-conotoxin MVIIC (1 μM), and L-type Ca_V_ channels with nifedipine (10 μM).

### Statistical analysis

Quasi-static lung compliance, BAL protein and cell counts, LIS values, cytokine concentrations, and FLIPR and Fluo-4 data are represented as Box-Whisker plots with median values, 1st and 3rd quartiles, and maximum and minimum values. FLIPR curves in Figs. 5A,B, 6A,B, and 7A,B show mean + SEM values. Data were analyzed using the unpaired student t-test, multivariate analysis of variance (ANOVA), and pairwise comparison of means using the Tukey–Kramer method to adjust for multiple comparisons. All statistical analyses were performed using GraphPad Prism 7 software (version 6.04, La Jolla, CA; https://www.graphpad.com/), and *p* values *p* ≤ 0.05 were considered significant.

### Study approval

Approval for all experiments was obtained from the “University of California Los Angeles Animal Research Committee (ARC). All experiments were performed in accordance with our institutional protocols, guidelines and recommendations.

## Supplementary information

Supplementary Legends.

Supplementary Figure 1.

Supplementary Figure 2.
