# Supplementary material for: K2P2.1 (TREK-1) potassium channel activation protects against hyperoxia-induced lung injury
Source: Sci Rep. 2020 Dec 15;10:22011. doi: 10.1038/s41598-020-78886-y (PMC7738539; doi:10.1038/s41598-020-78886-y)
Supplement: Supplementary file 1 — Supplementary Legends. [file 41598_2020_78886_MOESM1_ESM.docx]

**SUPPLEMENTARY MATERIAL**

**K_2P_2.1 (TREK-1) Potassium Channel Activation Protects Against Hyperoxia-Induced Lung Injury.**

Tatiana Zyrianova^1^, Benjamin Lopez^1^, Riccardo Olcese^2,3^, John Belperio^4^, Christopher M. Waters^5^, Leanne Wong^1^, Victoria Nguyen^1^, Sriharsha Talapaneni^1^, and Andreas Schwingshackl^1^.

^1^Departments of Pediatrics, ^2^Anesthesiology & Perioperative Medicine, ^3^Physiology, and ^4^Pulmonary & Critical Care Medicine, University of California Los Angeles, CA, USA.

^5^Department of Physiology, University of Kentucky, KY, USA.

Corresponding author:

Andreas Schwingshackl, M.D.; Ph.D.

University of California Los Angeles

Department of Pediatrics

10833 Le Conte Ave, MDCC 12-475, CA 90095

Email: [aschwingshackl@gmail.com](mailto:aschwingshackl@gmail.com)

Phone: 310-825-6752

**LEGENDS FOR SUPPLEMENTARY FIGURES**

**Supplementary Figure 1:**

Downregulation of TREK-1 gene and protein expression in primary mouse AT2 cells after 24 hours HO exposure, as shown by real-time PCR **(A)** and immunofluorescence (IF) microscopy **(B)**, respectively. PCR data are normalized to the housekeeping gene GAPDH; n=3, ^p=0.02, compared to room air-treated cells. The IF picture is representative of 3 separate experiments.

**Supplementary Figure 2:**

Dose-response curves for BL1249 and ML335 showing lack of toxicity of both compounds on primary human alveolar epithelial cells (HAEC) using CCK-8 and XTT cell viability assays, except with the highest ML335 dose tested; n=4, *compared to untreated vehicle control, p<0.05.
