# Supplementary figures and images for: K2P2.1 (TREK-1) potassium channel activation protects against hyperoxia-induced lung injury
Source: Sci Rep. 2020 Dec 15;10:22011. doi: 10.1038/s41598-020-78886-y (PMC7738539; doi:10.1038/s41598-020-78886-y)

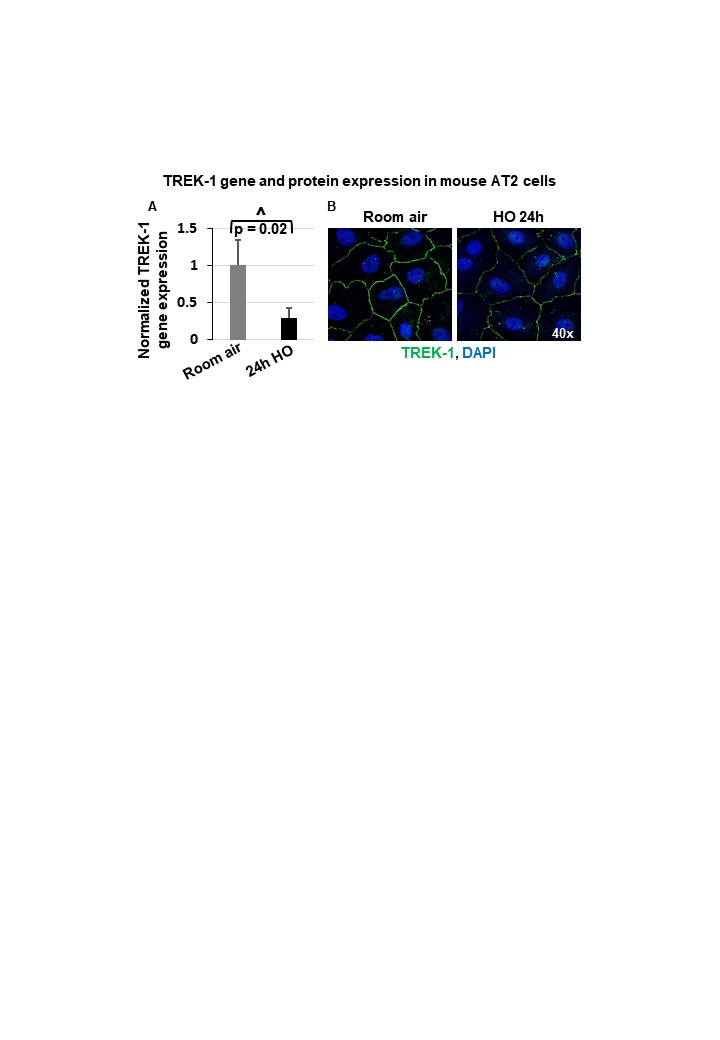

Supplement: Supplementary file 2 — Supplementary Figure 1. [file 41598_2020_78886_MOESM2_ESM.jpg]

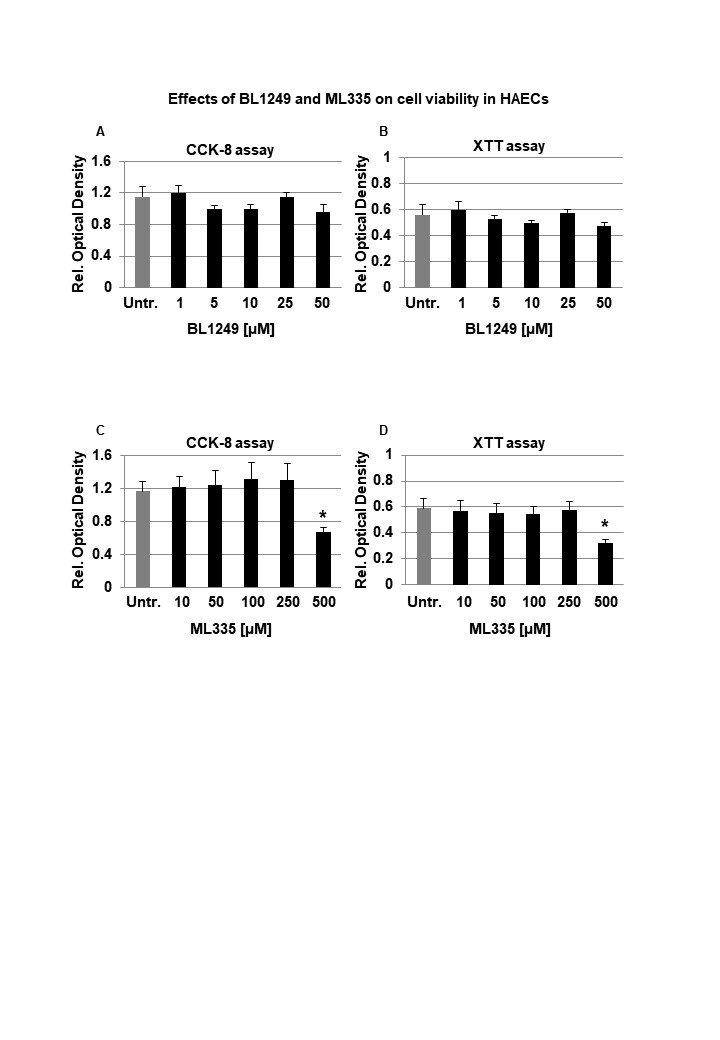

Supplement: Supplementary file 3 — Supplementary Figure 2. [file 41598_2020_78886_MOESM3_ESM.jpg]
